# *Plasmodium vivax* tryptophan-rich antigen reduces type I collagen secretion via the NF-κBp65 pathway in splenic fibroblasts

**DOI:** 10.1186/s13071-024-06264-y

**Published:** 2024-05-27

**Authors:** Wei-Zhong Kong, Hang-Ye Zhang, Yi-Fan Sun, Jing Song, Jian Jiang, Heng-Yuan Cui, Yu Zhang, Su Han, Yang Cheng

**Affiliations:** 1 Laboratory of Pathogen Infection and Immunity, Department of Public Health and Preventive Medicine, Wuxi School of Medicine, Jiangnan University, Wuxi, 214000 China; 2 Case Room, Sir Run Run Shaw Hospital, Zhejiang University School of Medicine, Hangzhou, 310016 China; 3 Department of Laboratory Medicine, Affiliated Hospital of Jiangnan University, Wuxi, Jiangsu China; 4 School of Food Science and Technology, Jiangnan University, Wuxi, Jiangsu People’s Republic of China; 5 Department of Obstetrics and Gynecology, Affiliated Hospital of Jiangnan University, Wuxi, China; 6 Wuxi Red Cross Blood Center, Wuxi, 214000 China; 7 Wuxi School of Medicine, Jiangnan University, Wuxi, 214000 China

**Keywords:** *Plasmodium vivax*, PvTRAgs, Spleen fibroblasts, NF-κBp65 pathway, Collagen

## Abstract

**Background:**

The spleen plays a critical role in the immune response against malaria parasite infection, where splenic fibroblasts (SFs) are abundantly present and contribute to immune function by secreting type I collagen (collagen I). The protein family is characterized by *Plasmodium vivax* tryptophan-rich antigens (PvTRAgs), comprising 40 members. PvTRAg23 has been reported to bind to human SFs (HSFs) and affect collagen I levels. Given the role of type I collagen in splenic immune function, it is important to investigate the functions of the other members within the PvTRAg protein family.

**Methods:**

Protein structural prediction was conducted utilizing bioinformatics analysis tools and software. A total of 23 PvTRAgs were successfully expressed and purified using an *Escherichia coli* prokaryotic expression system, and the purified proteins were used for co-culture with HSFs. The collagen I levels and collagen-related signaling pathway protein levels were detected by immunoblotting, and the relative expression levels of inflammatory factors were determined by quantitative real-time PCR.

**Results:**

In silico analysis showed that *P. vivax* has 40 genes encoding the TRAg family. The C-terminal region of all PvTRAgs is characterized by the presence of a domain rich in tryptophan residues. A total of 23 recombinant PvTRAgs were successfully expressed and purified. Only five PvTRAgs (PvTRAg5, PvTRAg16, PvTRAg23, PvTRAg30, and PvTRAg32) mediated the activation of the NF-κBp65 signaling pathway, which resulted in the production of inflammatory molecules and ultimately a significant reduction in collagen I levels in HSFs.

**Conclusions:**

Our research contributes to the expansion of knowledge regarding the functional role of PvTRAgs, while it also enhances our understanding of the immune evasion mechanisms utilized by parasites.

**Graphical Abstract:**

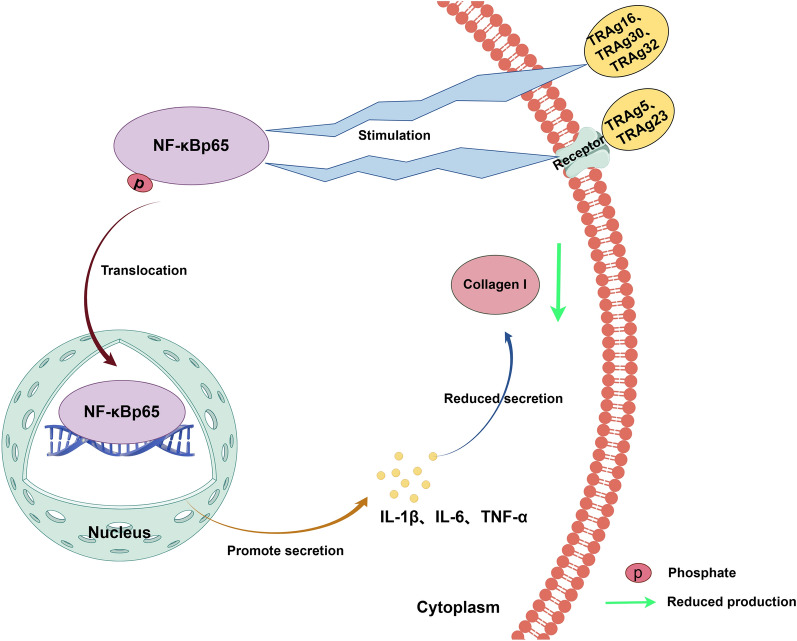

**Supplementary Information:**

The online version contains supplementary material available at 10.1186/s13071-024-06264-y.

## Background

Malaria is a devastating infectious disease, which imposes an immense worldwide health burden [1]. Among the malaria pathogens, *Plasmodium vivax* has the largest geographical distribution and the greatest economic impact [2–4]. Basic research on *P. vivax* has been impeded by the lack of a continuous in vitro culture system, unlike the well-established continuous in vitro culture system for *Plasmodium falciparum* [5, 6]. Although *P. vivax* has been historically described as “benign” malaria, *P. vivax* malaria is increasingly recognized as a cause of severe morbidity and mortality [7, 8]. Individuals infected with *P. vivax* develop various clinical symptoms, such as fever, anemia, splenomegaly, or severe malaria [9]. However, the pathogenesis of severe vivax syndromes remains poorly understood and requires further research [10].

The spleen, which is the largest immune organ, is mainly involved in filtering the blood, capturing pathogens, and activating the adaptive immune response [11]. *Plasmodium vivax* infection can lead to significant changes in the spleen, which cause asymptomatic swelling or complications such as rupture and hypersplenism [12, 13]. The splenic fibroblasts (SFs) are essential for maintaining the structure and immune function of the spleen [14]; these are found extensively in connective tissue and secrete collagen I into the extracellular matrix (ECM) [15, 16]. Collagen I is the main component of ECM, which is critical for creating the microenvironment needed for immune response development [17, 18]. Furthermore, the collagen network contributes to the proper functioning of the immune system of the spleen [19]. In the spleen, SFs secrete and remain associated with collagen I; they combine with argyophilic reticular fibers, which strengthen the filtration beds [20]. The central function of the spleen—selective clearance of cells, microbes, and other particles from the blood—depends upon these filtration beds. Such functions of the spleen as phagocytosis and immunologic reactivity derive from clearance capacity of filtration beds [21]. In rodents infected with malaria parasites, iRBCs circulate in the bloodstream and pass through the spleen filtration bed, where they are diverted from arterioles to venous sinuses and eventually cleared by immune cells in the filtration bed [19], while the specific circulation and clearance mechanism of iRBCs in the human spleen remains unclear. Upon infection with *Plasmodium*, SFs are aberrantly activated and then evolve into barrier cells that interact with fibronectin and collagen I to form the blood-spleen barrier [22, 23]. The barrier cells protect RBCs from destruction by parasites but allow iRBCs to adhere and realize immune escape, which can sometimes lead to an imbalance in the immune response and even to severe disease [24, 25]. Thus, it is important to understand the changes in SFs and collagen I during *Plasmodium* infection.

The interaction between the malaria parasite and host cells mediated through ligand-receptor binding, especially involving malaria parasite export proteins, plays essential roles [26–28]. Malaria parasite export proteins are transported to the membrane surface of infected erythrocytes, which enhances the adhesion of infected erythrocytes to host cells and facilitates immune evasion by the parasite [29]. *Plasmodium falciparum* erythrocyte membrane protein 1 (PfEMP1) is expressed on the surface of mature infected erythrocytes and binds to endothelial cells, which allows evasion from circulation and destruction in the spleen [30, 31]. The VIR14 protein of the *P. vivax vir* gene family can be transported to the surface of reticulocytes and adheres to splenic fibroblasts (SFs) through the ICAM-1 receptor [23].

Similarly, the protein characterized by *P. vivax* tryptophan-rich antigens (PvTRAgs), as export protein, can be delivered to the surface of the erythrocyte membrane to bind with host cells [32, 33]. Recently, PvTRAg5 has been found to bind to erythrocyte receptors basigin and band 3 to facilitate parasite growth [34]. PvTRAg23 was found to interact with human SFs (HSFs) and reduce collagen I levels [35]. The reduced collagen I levels may potentially affect the hemofiltration function of the spleen, which facilitates parasite evasion. However, determining whether all PvTRAgs participate in changes in collagen I is interesting because of the elusive precise functions of PvTRAgs. Notably, there are 36 members of the PvTRAg family in the *P. vivax* reference genome Sal-I and 40 members of the PvTRAg family in the *P. vivax* reference genome PvP01, PvPAM, and PvW1.

This study aimed to identify the interactions between the PvTRAgs and SFs and assess their effect on collagen I levels in HSFs. Here, the activation of NF-κBp65 pathway results in the upregulation of inflammatory factor expression in response to PvTRAgs stimulation, which induces a decrease in collagen I levels. Our study expands the landscape of the functional roles of PvTRAgs and raises interesting hypotheses for the broader *Plasmodium* TRAg gene family.

## Methods

### Bioinformatics analysis

To acquire the information of *pvtrag* gene structure, the genes encoding *pvtrag* were retrieved from PlasmoDB (https://plasmodb.org/, PlasmoDB ID in Additional file 1: Table S1). The Simple Modular Architecture Research Tool (SMART) (http://smart.embl-heidelberg.de/) and the GPI Fungal Prediction Server (https://mendel.imp.ac.at/gpi/cgi-bin/gpi_pred_fungi.cgi) were used for the prediction of signal peptides and glycophosphatidylinositol-anchored (GPI-anchored) proteins.

### PCR amplifcation of *pvtrag*

To amplify 36 *pvtrag* genes, genomic DNA of the *P. vivax* Sal-I strain was extracted. The primers used for amplification are described in Additional file 2: Table S1, and the genomic DNA was extracted as previously described [36]. The PCR reactions were conducted in a 10-μl reaction volume consisting of 1 μl genomic DNA as the template, 0.5 μl of each primer (5 μM), 2 μl 5 × TransStart^®^ FastPfu Bufer (TransGen Biotech Co., Ltd., Beijing, China), 0.8 μl dNTPs (2.5 mM), 0.25 μl FastPfu DNA Polymerase (TransGen Biotech Co., Ltd.), and 4.95 μl nuclease-free water. The PCR amplification was performed in a Mastercycler (Eppendorf, Hamburg, Germany) under the following program: denaturation at 95 ℃ for 2 min, followed by 35 cycles of 95 ℃ for 20 s, 50 ℃ for 30 s, and 72 ℃ for 1 min, and a final extension at 72 ℃ for 5 min. The sizes of the PCR products were estimated using the (*Trans2K* Plus DNA marker (TransGen Biotech Co., Ltd.). All PCR products were sequenced by YiXin Biotechnology (Shanghai, China).

### Expression and purification of recombinant proteins

To obtain recombinant PvTRAg proteins, *Escherichia coli* BL21 (DE3) pLysS cells (TransGen Biotechnology, Beijing, China) were used to express the corresponding proteins. Briefly, the *pvtrag* genes were amplified by PCR and cloned into the pET30 vector (Tianlin Bio, Wuxi). This vector adds six-histidine tags at the N- and C-terminal ends, which enables easier purification and immunodetection using monoclonal antibodies against the six-histidine tag. Next, the recombinant plasmids were transformed into *E. coli* BL21 (DE3) pLysS cells; 200 ml Luria-Bertani (LB) medium supplemented with 100 mg/ml ampicillin was inoculated with a transformed bacterial pre-culture and shaken at 37 ℃ until the cell density reached an OD600 of approximately 0.6–0.8; protein expression was induced with 0.1 mM isopropyl-β-D-thiogalactoside (IPTG) at 16 ℃ for 18–20 h [37]. Then, culture effluent was collected, and cells were harvested by centrifugation at 300 × *g* for 30 min at 4 ℃. The rPvTRAg proteins were purified under nondenaturing conditions by a biotechnology company (Youlong Bio, Shanghai, China) using a Ni agarose column. PvTRAgs were mixed with the protein loading buffer, boiled at 100 ℃ for 5 min, and separated in 10% sodium dodecyl sulfate-polyacrylamide gel electrophoresis (SDS-PAGE) gels. Next, they were stained with Coomassie Brilliant Blue (R250). For immunoblotting, PvTRAgs were detected using horseradish peroxide (HRP)-coupled anti-His antibody (1:5000, Abcam, Cambridge, MA, USA).

### Cell culture

HSFs (ScienCell, CA, USA) were cultured in fibroblast medium (ScienCell, USA) containing 1% fibroblast growth supplement, 2% fetal bovine serum, and 1% antibiotic solution (P/S) at 37 ℃ in a humidified incubator containing 5% CO_2_ [38]. Prior to culturing HSFs, dishes need to be coated with polylysine (ScienCell, USA) for 2 h at room temperature to increase cell adhesion. The plates were rinsed twice with sterilized ultrapure water, and 8 ml complete medium was added to culture the cells. For cell passaging, a 95% fusion of cells is required.

### Western blot analysis

HSFs were plated in 12-well culture plates and incubated at 37 ℃ in a cell incubator. When the cell density was about 70%, medium was removed and fresh medium containing PvTRAgs was added.

To analyze the binding capacity of PvTRAgs to HSFs, PvTRAgs were co-incubated with HSFs for 4 h. To assess the effects of PvTRAgs on collagen I synthesis, PvTRAgs were co-incubated with HSFs for 48 h. To examine the activation of cellular signaling, HSFs were treated with PvTRAgs for 0.5 h. The medium was discarded, and cells were washed once with ice-cold PBS after the experimental time period. Cells were then collected and resuspended in cold RIPA lysis buffer (Beyotime, China) containing protease and phosphatase inhibitors. Protein concentration was determined by BCA assay (Beyotime, China). Then, total protein (10–20 µg) was mixed with protein loading buffer, and all samples were boiled and separated on 10% SDS-PAGE. The separated proteins were transferred to polyvinylidene difluoride membranes. The membranes were blocked with closure buffer (1 × TBST, 5% milk powder) for 2 h at room temperature. They were then incubated with anti-His (1:1000, Abcam, Cambridge, MA, USA), anti-collagen I (1:1000, Abcam), anti-NF-κBp65 (1:1000, CST, Danvers, MA, USA), anti-phospho-NF-κBp65 (1:1000, CST), anti-FAK (1:1000, CST), anti-phospho-FAK (1:1000, CST), anti-p38 MAPK (1:1000, CST), anti-phospho-p38 MAPK (1:1000, CST), or anti-GAPDH (1:1000, Abcam) at 4 ℃ overnight. After washing with TBST, the membrane was incubated for 1 h with secondary antibody conjugated to HRP (1:5000, Abcam, Cambridge, USA). Bands were visualized by an ECL detection kit (XINSAIMEI, Suzhou, China), and protein expression was quantified with Image J (National Institutes of Health, MD, USA). Analysis of WB results was conducted using Image J software.

### RNA extraction and quantitative real-time PCR

To examine the levels of cellular inflammatory factor mRNA, qPCR was performed as previously described [35]. PvTRAgs were co-incubated with HSFs after 48 h total RNA had been isolated from HSFs in accordance with the protocols of the manufacturer (Yeasen Biotechnology, Shanghai, China). The extracted total RNA was further processed to remove genomic DNA and then reverse transcribed into complementary DNA (cDNA) using 4 × HiFiScript RTMaster Mix, following the instructions for the RNA Reverse Transcription Kit (Yeasen Biotechnology, Shanghai, China). RT-PCR was performed in triplicate with three independent samples for each experimental group in a LightCycler480 II apparatus (Roche, USA) with SYBR Green Master Mix (No Rox) (Yeasen Biotechnology, Shanghai, China). The amplification program followed a two-step method. Thermal cycling conditions were as follows: pre-denaturation at 95 ℃ for 5 min, followed by 40 PCR cycles at 95 ℃ for 10 s and 60 ℃ for 30 s. The ratio of each target gene was determined using GAPDH as an internal control. The relative expression levels of genes were calculated from the quantification cycle (Cq) value and standardized by the 2^−ΔΔCq^ method. Primers for amplified genes (IL-1β, IL-6, and TNF-α) are presented in Additional file 2: Table S2.

### Statistical analysis

Immunoblotting and qRT-PCR analyses were repeated three times (*n* = 3). All statistical analyses were performed using GraphPad Prism 9.5 software (GraphPad, San Diego, CA, USA). Data from two or more groups were compared using one-way ANOVA, and differences between the two groups were analyzed using t-test. *P* < 0.05 was considered statistically significant (*P < 0.05, **P < 0.01, ***P < 0.001, ****P < 0.0001).

## Results

### Expression and purification of PvTRAgs

**Fig.1:**
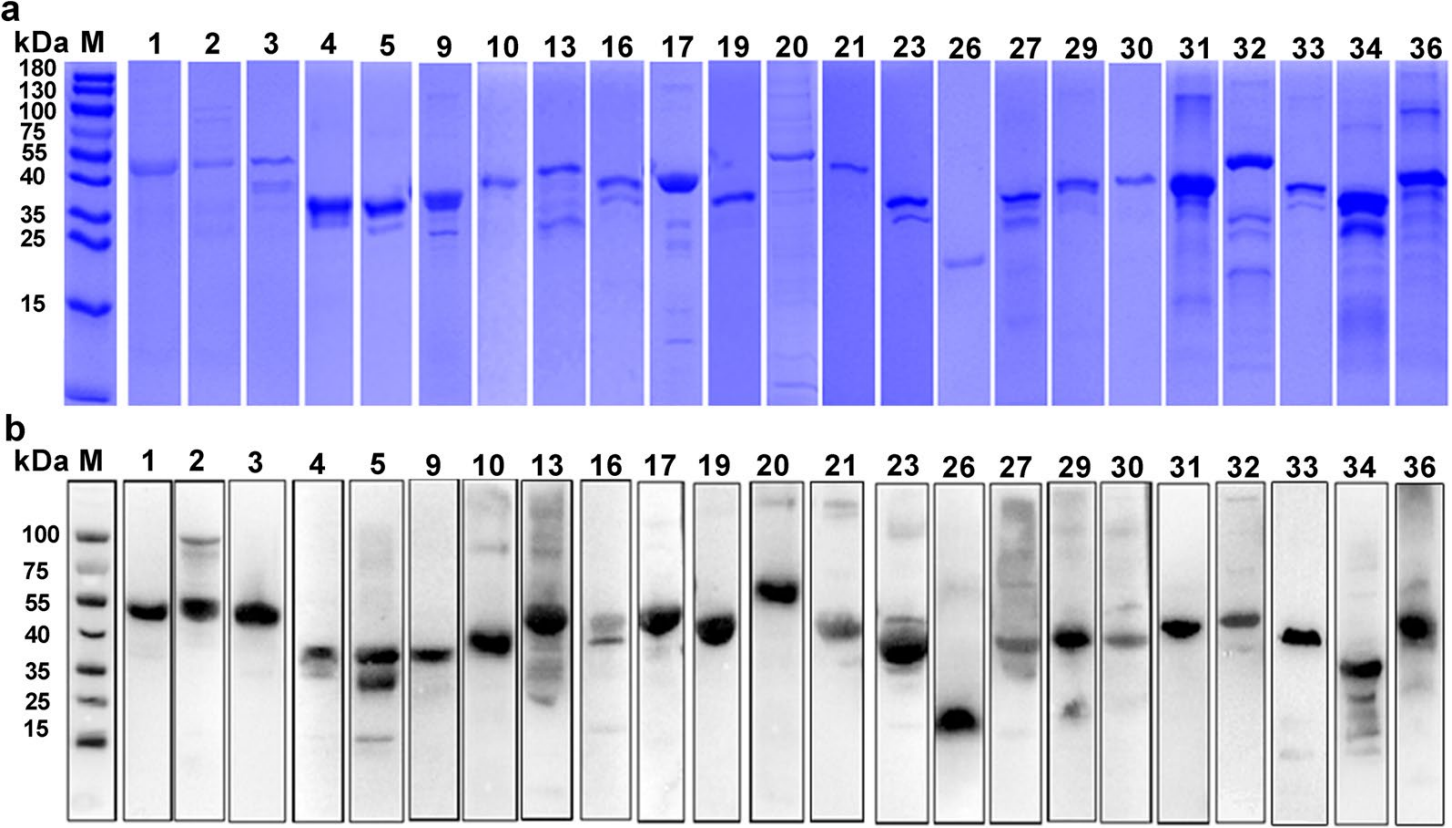
SDS-PAGE and Western blot analysis of purified His-tag recombinant PvTRAgs. **a** SDS-PAGE analysis of purified PvTRAgs was performed under denaturing and reducing conditions. Gel protein content was detected by Coomassie blue staining. **b** Western blot analysis with anti-His antibody was performed to confirm the expression of His-tag on the recombinant protein. The molecular weight of the protein band includes the size of 6X His-tag. M: Protein marker; 1,2…36: PvTRAg proteins

We summarized the structural features of PvTRAgs to investigate their molecular function. The smallest protein in the PvTRAg protein family was PvTRAg26 (223 amino acids; 26 kDa), and the largest protein was PvTRAg18 (2662 amino acids; 309 kDa). All PvTRAg proteins contain a tryptophan-rich structural domain in the C-terminal region. Characterization information for all members of the PvTRAgs is presented in Additional file 3: Table S3. We attempted to express and purify the recombinant PvTRAgs (rPvTRAgs) from *E. coli* to further characterize PvTRAgs function. Fusion proteins were successfully expressed for 23 of the 36 clones. The rPvTRAgs were purified under nondenaturing conditions (Fig. 1a). Corresponding immunoblotting assays were performed with anti-His tag monoclonal antibodies (Fig. 1b).

### rPvTRAg treatment leads to different changes in collagen I levels in HSFs

**Fig.2:**
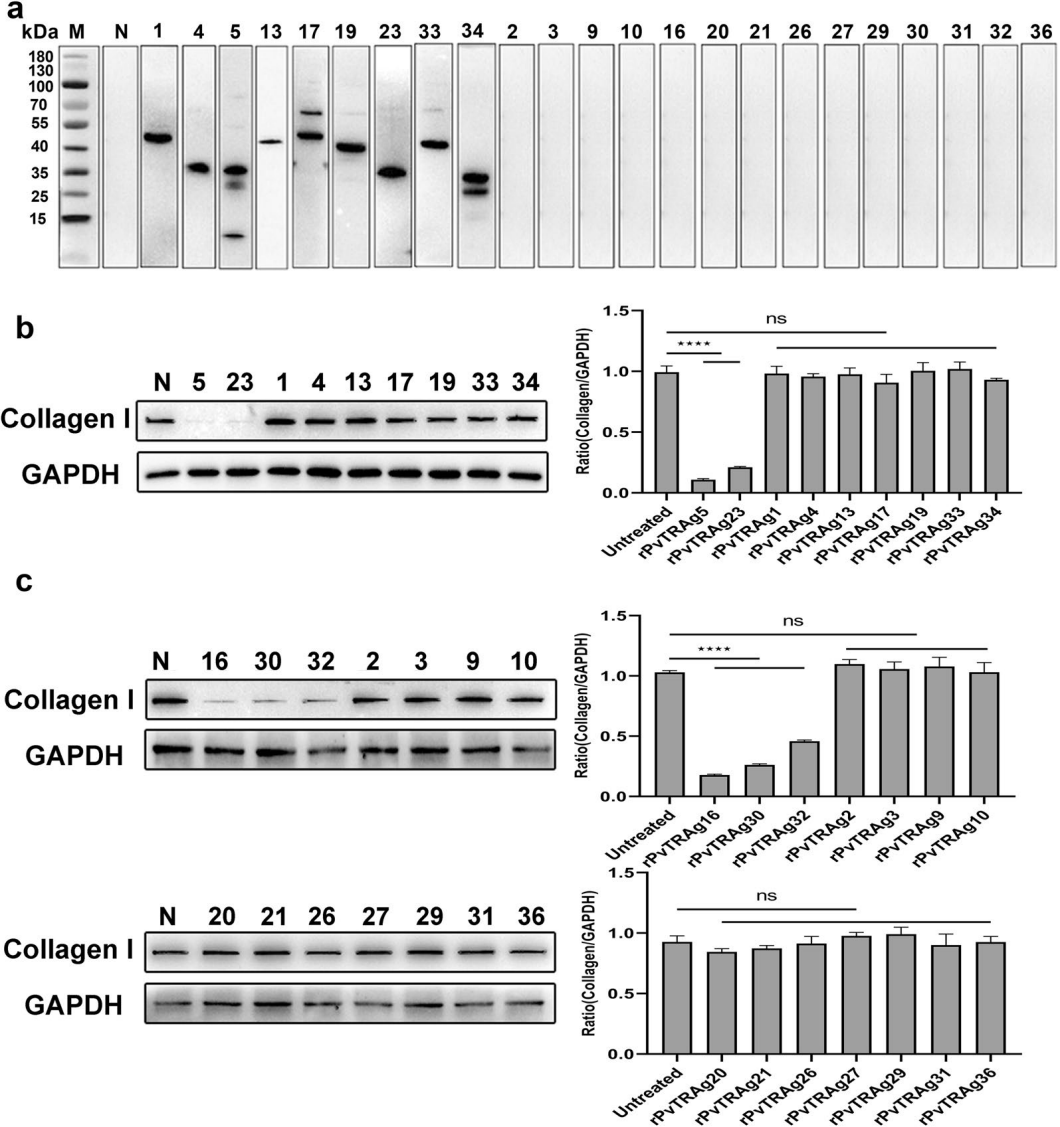
The levels of collagen I secreted by HSFs after stimulated by recombinant PvTRAgs. **a** Screening results of PvTRAg proteins binding to HSFs; **b** 5 × 10^6^ HSFs were incubated with bindable recombinant proteins at the corresponding concentrations. After 48 h, the cells were lysed and the expression of collagen I in HSFs was detected by immunoblotting (left), and the gray values were compared between the two groups using GAPDH as an internal reference (right); **c** 5 × 10^6^ HSFs were incubated with unbindable recombinant proteins at the corresponding concentrations. After 48 h, the cells were lysed and the expression of collagen I in HSFs was detected by immunoblotting, and the gray values were compared between the two groups using GAPDH as an internal reference. M: Protein marker; N: untreated HSF cells; 1,4…36: PvTRAg proteins. Statistical analyses were carried out by one-way ANOVA with Dunnett’s multiple comparisons test (ns *P* ≥ 0.05; *P < 0.05; **P < 0.01, ***P < 0.001, ****P < 0.0001)

The successfully expressed rPvTRAgs were co-incubated with HSFs for 4 h to explore the interaction between PvTRAgs and HSFs. Nine PvTRAgs (PvTRAg1, PvTRAg4, PvTRAg5, PvTRAg13, PvTRAg17, PvTRAg19, PvTRAg23, PvTRAg33, and PvTRAg34) were identified as capable of binding to HSFs (Fig. 2a).

HSFs were treated with rPvTRAgs known to bind with cells for 48 h to explore whether other PvTRAgs interacting with HSFs affect collagen I. The results showed that only PvTRAg5 and PvTRAg23 significantly reduced the collagen I levels in HSFs (*P* < 0.0001, Fig. 2b). This result suggests that not all PvTRAgs (binding to HSFs) resulted in a decrease in collagen I levels.

HSFs were treated with rPvTRAgs (without binding to HSFs) to explore whether rPvTRAgs would act directly on HSFs. Despite certain proteins in the PvTRAg family not binding to HSFs, PvTRAg16, PvTRAg30, and PvTRAg32 also significantly reduced collagen I levels (*P* < 0.0001, Fig. 2c). These results provide strong evidence that PvTRAgs can specifically signal HSFs to decrease collagen I independent of receptor and ligand anchoring.

### Type I collagen downregulation is linked to NF-κB signaling pathway activation

**Fig. 3:**
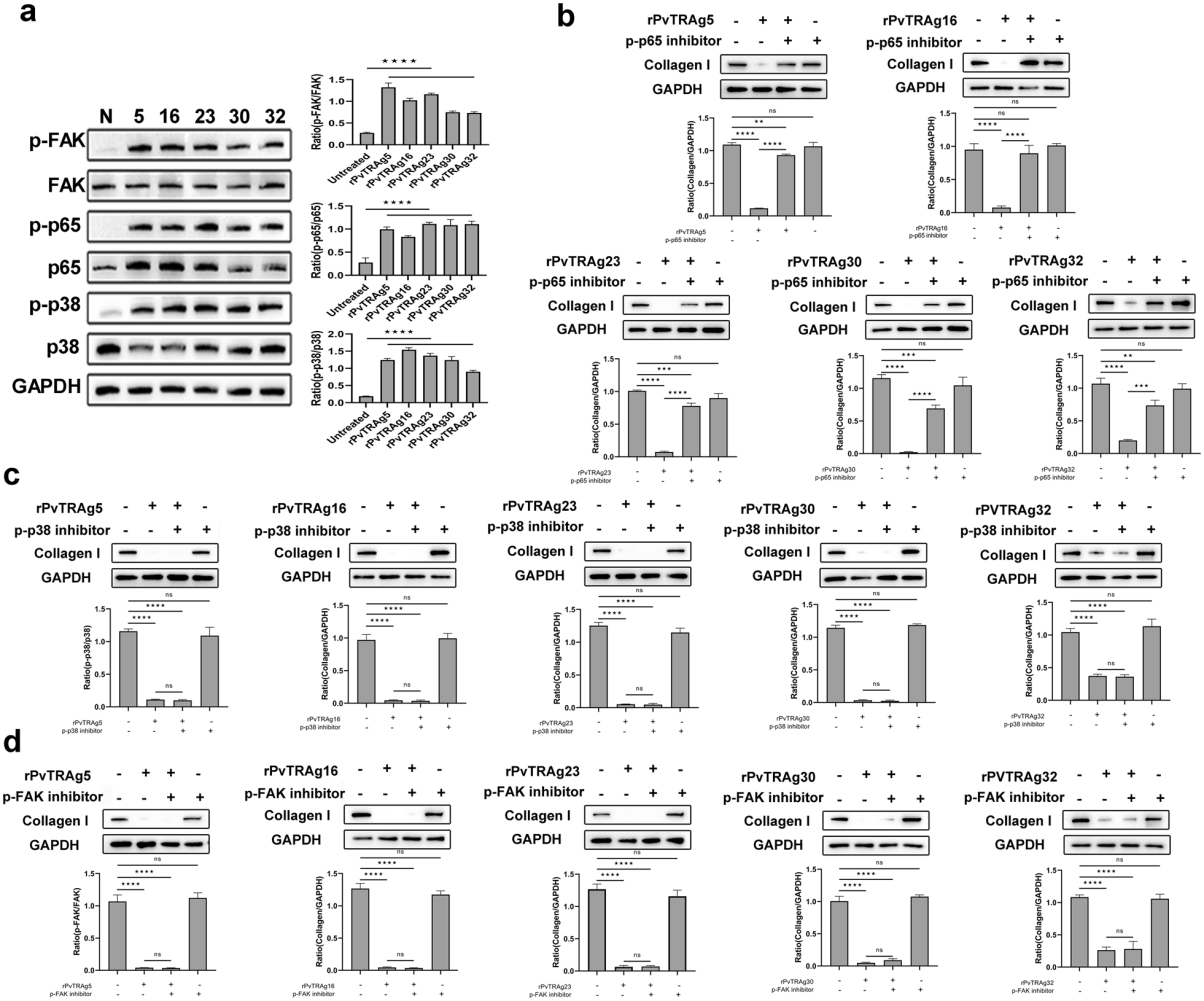
NF-κBp65 pathway mediates decreased collagen I. **a** The 5 × 10^6^ HSFs were treated with 50 mg/ml rPvTRAg5, rPvTRAg16, rPvTRAg23, rPvTRAg30, and rPvTRAg32, and the treated cells were collected and incubated with the corresponding signaling pathway antibodies, respectively. The activation status of each group of pathways was detected by immunoblotting, and GAPDH was used as an internal reference. **b** HSFs were pretreated with the NF-κBp65 signaling pathway inhibitors for 1 h; **c** HSFs were pretreated with the FAK or P38 MAPK signaling pathway inhibitors for 1 h; 50 mg/ml rPvTRAg5, rPvTRAg16, rPvTRAg23, rPvTRAg30, and rPvTRAg32 stimulated the treated and untreated cells; 48 h later, the expression of collagen I was detected in each group by immunoblotting, and the comparison was made by using GAPDH as an internal involved group. Plus (+) indicates added; minus (−) indicates not added

HSFs were treated with rPvTRAgs (PvTRAg5, PvTRAg16, PvTRAg30, and PvTRAg32) for 0.5 h to investigate the signaling pathway responsible for the reduction in collagen I levels. The results showed that three signaling pathways (NF-κBp65, FAK, and P38 MAPK) were activated after stimulation by PvTRAg5, PvTRAg16, PvTRAg30, and PvTRAg32 (Fig. 3a). HSFs were treated with appropriate signaling pathway inhibitors (Bay 11–7082, SB203580, and TAE226) to identify which pathways mediate the reduction in collagen I levels. After 1 h inhibitor treatment, HSFs were subsequently co-incubated with rPvTRAgs for 48 h. The results showed that collagen I levels increased after inhibition of the NF-κBp65 pathway, whereas they decreased after inhibition of the other pathways compared with the control group. Notably, the results are consistent with those for four proteins mentioned above (Fig. 3b–d). Therefore, only the NF-κBp65 pathway was found to be responsible for the decrease in collagen.

### Expression of IL-1β, IL-6, and TNF-α decreases type I collagen in HSFs

The HSFs were treated with PvTRAgs for 48 h (PvTRAg5, PvTRAg16, PvTRAg30, and PvTRAg32) to assess the levels of inflammatory factors. Meanwhile, HSFs stimulated with PvTRAg1 (bound to HSFs) and PvTRAg2 (not bound to HSFs) were used as control groups.

**Fig.4:**
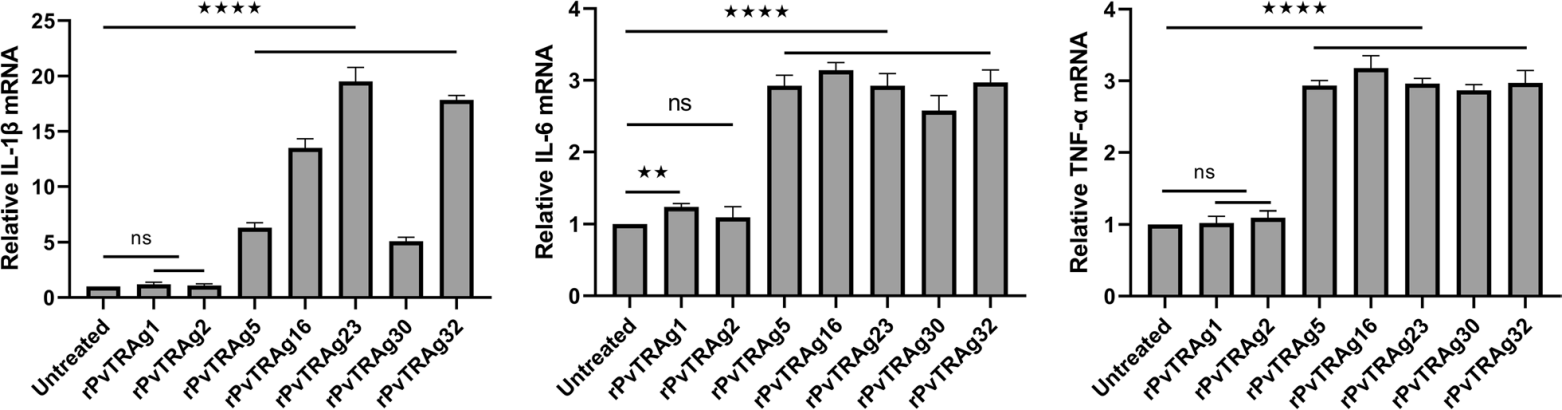
Elevated expression of IL-1β, IL-6, and TNF-α in HSFs. 5 × 10^6^ HSFs were treated with 50 mg/ml rPvTRAg1, rPvTRAg2, rPvTRAg5, rPvTRAg16, rPvTRAg23, rPvTRAg30, and rPvTRAg32, respectively, and RNA was extracted from treated and untreated groups after 48 h. The expression of the relevant cytokines was detected by qPCR and compared with that of the control group. Three independent experiments have been performed. Statistical analyses were carried out by one-way ANOVA with Dunnett’s multiple comparisons test (ns *P* ≥ 0.05; *P < 0.05; **P < 0.01, ***P < 0.001, ****P < 0.0001)

The results showed that IL-1β, IL-6, and TNF-α levels were significantly higher in the groups simulated with rPvTRAg5, rPvTRAg16, rPvTRAg23, rPvTRAg30, and rPvTRAg32 than in the control group (*P* < 0.0001, Fig. 4). Overall, PvTRAgs (PvTRAg5, PvTRAg16, PvTRAg23, PvTRAg30, and PvTRAg32) activated the NF-κB p65 signaling pathway in HSFs. This activation triggered an inflammatory response, which ultimately led to a decrease in the collagen I levels.

## Discussion

TRAgs have been studied in a wide range of *Plasmodium* spp., but a clear molecular function for these antigens has not been determined [39]. *Plasmodium vivax* exhibits a higher abundance of TRAgs than other human malaria parasites, and their occurrence in clusters along chromosomes suggests expansion through gene duplication and diversification. Gene expansion is always accompanied by functional diversification [40]. Previously found stage-specific expression of *pvtrag* genes confirms their functional diversification in the life cycle of parasites [36]. Notably, PvTRAg23 has been identified to alter collagen I levels in HSFs. Therefore, we aimed to characterize 23 PvTRAgs by examining their ability to bind to HSFs and their impact on collagen levels. Our findings demonstrate that PvTRAgs can specifically reduce collagen I levels by activating the NF-κB p65 signaling pathway.

Binding of merozoite proteins to host cells is critical for their invasion, immune evasion [41, 42], and damage to host cells and tissues [43]. After infection of human erythrocytes, malaria parasite export proteins are transported to the iRBC membrane and act as mediators for interactions with host cells [44]. Similarly, PvTRAgs exhibit strong immunogenicity and partial binding to erythrocytes, which contributes to the pathogenesis of malaria [33]. Here, PvTRAgs were co-incubated with HSFs, and nine proteins were identified for binding to HSFs (Fig. 2a). Recent studies have shown that *P. vivax*-infected reticulocytes can adhere to HSFs via VIR14 and may avoid clearance by macrophages [23, 45]. Whether the binding of PvTRAg to HSFs contributes to *P. vivax* adhesion to HSFs remains to be determined and will not be discussed further here. Among the nine proteins proteins that could bind to HSFs, PvTRAg5 and PvTRAg23 decreased collagen I levels (Fig. 2b). This finding suggests that the receptor might not be structurally or functionally altered even in the presence of receptor–ligand interaction. Expression of the *PvTRAg*23 gene was high in Brazilian isolates; however, it was relatively low in Cambodian and East African isolates [46]. The *PvTRAg*23 gene expressions vary between different *P. vivax* strains as a contributing factor to antigenic variation [46–48], which may favor different immune evasion strategies for different *P. vivax* strains [49].

Although the specific binding of receptors and ligands is fundamentally important for various cellular processes and biochemical activities [50, 51], some proteins do not act through the formation of receptor–ligand complexes [52, 53]. Non-adhesive PvTRAgs may also play a crucial role in parasite growth [33]. The results show that three PvTRAgs (PvTRAg16, PvTRAg30, PvTRAg32) can directly act on HSFs to reduce the collagen I levels (Fig. 2c). These results indicate that the members of the PvTRAg protein family regulate collagen I levels through direct and indirect interactions with HSFs, which lead to a reduction in collagen I levels.

Fibroblasts produce large amounts of collagen I, which is the most abundant component of the ECM [15, 54]. The ECM can perform microbial recognition and phagocytosis in the immune response to infection [55] and can convey specific signals to immune cells encountering or navigating through it [56]. Furthermore, the collagen network contributes to the proper functioning of the immune system in the spleen [19, 20]. Overall, reduced expression of collagen I leads to altered ECM, which affects ECM-mediated functions in the spleen. Therefore, a decrease in collagen I levels by PvTRAgs can influence immune functions in the spleen, such as blood filtration and trapping of pathogens, which may ultimately contribute to parasite escape. Recently, a study has unequivocally shown that the spleen sustains a very large biomass of non-phagocytosed asexual *P. vivax* parasites and *P. vivax* malaria is mainly a cryptic erythrocytic infection of the spleen [57, 58]. Impaired splenic blood clearance contributes to parasite immune evasion and may favor the accumulation of malaria parasites in the spleen to enhance malaria parasite survival and replication in the spleen.

Altered cellular collagen levels are frequently accompanied by the activation of the NF-κBp65, FAK, and P38 MAPK signaling pathways [59–61]. PvTRAg23 mediates the activation of the NF-κBp65 signaling pathway, which leads to the upregulation of IL-1β, IL-6, and TNF-α [35]. This phenomenon inhibits the collagen I levels in HSFs [35]. In this study, the four other PvTRAgs, namely, PvTRAg5, PvTRAg16, PvTRAg30, and PvTRAg32, were also found to be involved in the reduction in collagen I levels in HSFs. The reduction in collagen I levels was determined to be mediated by the NF-κBp65 signaling pathway (Fig. 3a–c). Elevated levels of relevant inflammatory factors (IL-1β, IL-6, and TNF-α) were observed, as expected (Fig. 4). IL-1β, IL-6, and TNF-α have been found to inhibit the production of type I collagen [35]. The same situation was present in diabetic rat cardiomyocytes, where levels of TNF-α, IL-6, and IL-1b were negatively correlated with mRNA expression of collagen I [62]. Therefore, these inflammatory cytokines play a role in the regulation of collagen expression.

Despite our discovery of the function for PvTRAgs, some limitations need further improvement, which lays the groundwork for the exploration of the molecular functions of this unique family of *Plasmodium* protein. First, 13 recombinant PvTRAgs were unsuccessfully expressed. Thus, the functional characterization of these proteins needs further determination. Second, the receptor through which PvTRAgs bind to HSFs was not identified, and the role of ligand-receptor binding in causing a decrease in collagen I was unclear. Third, the activation mechanism of the NF-κBp65 signaling pathway by PvTRAgs was unknown.

## Conclusions

Five members of the PvTRAg family of proteins (PvTRAg5, PvTRAg16, PvTRAg23, PvTRAg30, and PvTRAg32) are capable of activating NF-κBp65 in HSFs, which subsequently results in an inflammatory response and ultimately a decrease in collagen I levels. The reduced collagen levels caused by PvTRAgs may impact spleen functions, such as clearance and filtration, which may ultimately facilitate the realization of parasite escape.

### Supplementary Information

**Additional file 1: Table S1.** Primers used for the amplification of *pvtrag* genes.

**Additional file 2: Table S2.** Sequences of the primers used for inflammatory factors.

**Additional file 3: Table S3.** Characterization information for all members of the PvTRAg family.

## Data Availability

The data supporting the conclusions of this article are included within the article and its additional file.

## Abbreviations

*P.vivax*: *Plasmodium vivax*
PvTRAgs: *Plasmodium vivax* tryptophan-rich antigens
SFs: Splenic fibroblasts
HSFs: Human splenic fibroblasts
Collagen I: Type I collagen
ECM: Extracellular matrix
IL-1β: Interleukin-1b
IL-6: Interleukin-6
TNF-α: Tumor necrosis factor-α

## References

[1] Baird JK (2013). Evidence and implications of mortality associated with acute *Plasmodium vivax* malaria. Clin Microbiol Rev.

[2] Battle KE, Gething PW, Elyazar IR, Moyes CL, Sinka ME, Howes RE (2012). The global public health significance of *Plasmodium vivax*. Adv Parasitol.

[3] Gething PW, Elyazar IR, Moyes CL, Smith DL, Battle KE, Guerra CA (2012). A long neglected world malaria map: *Plasmodium vivax* endemicity in 2010. PLoS Negl Trop Dis.

[4] Roth A, Maher SP, Conway AJ, Ubalee R, Chaumeau V, Andolina C (2018). A comprehensive model for assessment of liver stage therapies targeting *Plasmodium vivax* and *Plasmodium falciparum*. Nat Commun.

[5] Trager W, Jensen JB (1976). Human malaria parasites in continuous culture. Science.

[6] Noulin F, Borlon C, Van Den Abbeele J, D'Alessandro U, Erhart A (2013). 1912–2012: a century of research on *Plasmodium vivax* in vitro culture. Trends Parasitol.

[7] Blagborough AM, Musiychuk K, Bi H, Jones RM, Chichester JA, Streatfield S (2016). Transmission blocking potency and immunogenicity of a plant-produced Pvs25-based subunit vaccine against *Plasmodium vivax*. Vaccine.

[8] Mueller I, Galinski MR, Baird JK, Carlton JM, Kochar DK, Alonso PL (2009). Key gaps in the knowledge of *Plasmodium vivax*, a neglected human malaria parasite. Lancet Infect Dis.

[9] Bassat Q, Alonso PL (2011). Defying malaria: fathoming severe *Plasmodium vivax* disease. Nat Med.

[10] Kaur H, Sehgal R, Kumar A, Bharti PK, Bansal D, Mohapatra PK (2020). Distribution pattern of amino acid mutations in chloroquine and antifolate drug resistance associated genes in complicated and uncomplicated *Plasmodium vivax* isolates from Chandigarh, North India. BMC Infect Dis.

[11] Lewis SM, Williams A, Eisenbarth SC (2019). Structure and function of the immune system in the spleen. Sci Immunol.

[12] Imbert P, Rapp C, Buffet PA (2009). Pathological rupture of the spleen in malaria: analysis of 55 cases (1958–2008). Travel Med Infect Dis.

[13] Hamel CT, Blum J, Harder F, Kocher T (2002). Nonoperative treatment of splenic rupture in malaria tropica: review of literature and case report. Acta Trop.

[14] Bellomo A, Gentek R, Golub R, Bajenoff M (2021). Macrophage-fibroblast circuits in the spleen. Immunol Rev.

[15] Fu H, Zhang Y, An Q, Wang D, You S, Zhao D (2022). Anti-photoaging effect of Rhodiola rosea fermented by *Lactobacillus plantarum* on UVA-damaged fibroblasts. Nutrients.

[16] Shen R, Xu PP, Wang N, Yi HM, Dong L, Fu D (2020). Influence of oncogenic mutations and tumor microenvironment alterations on extranodal invasion in diffuse large B-cell lymphoma. Clin Transl Med.

[17] Tomasek JJ, Gabbiani G, Hinz B, Chaponnier C, Brown RA (2002). Myofibroblasts and mechano-regulation of connective tissue remodelling. Nat Rev Mol Cell Biol.

[18] Stellato M, Czepiel M, Distler O, Blyszczuk P, Kania G (2019). Identification and isolation of cardiac fibroblasts from the adult mouse heart using two-color flow cytometry. Front Cardiovasc Med.

[19] Ghosh D, Stumhofer JS (2021). The spleen: "epicenter" in malaria infection and immunity. J Leukoc Biol.

[20] Weiss L, Geduldig U, Weidanz W (1986). Mechanisms of splenic control of murine malaria: reticular cell activation and the development of a blood-spleen barrier. Am J Anat.

[21] Weiss L (1990). The spleen in malaria: the role of barrier cells. Immunol Lett.

[22] Weiss L (1989). Mechanisms of splenic control of murine malaria: cellular reactions of the spleen in lethal (strain 17XL) *Plasmodium yoelii* malaria in BALB/c mice, and the consequences of pre-infective splenectomy. Am J Trop Med Hyg.

[23] Fernandez-Becerra C, Bernabeu M, Castellanos A, Correa BR, Obadia T, Ramirez M (2020). *Plasmodium vivax* spleen-dependent genes encode antigens associated with cytoadhesion and clinical protection. Proc Natl Acad Sci U S A.

[24] Martin-Jaular L, Ferrer M, Calvo M, Rosanas-Urgell A, Kalko S, Graewe S (2011). Strain-specific spleen remodelling in *Plasmodium yoelii* infections in Balb/c mice facilitates adherence and spleen macrophage-clearance escape. Cell Microbiol.

[25] Bernabeu M, Lopez FJ, Ferrer M, Martin-Jaular L, Razaname A, Corradin G (2012). Functional analysis of *Plasmodium vivax* VIR proteins reveals different subcellular localizations and cytoadherence to the ICAM-1 endothelial receptor. Cell Microbiol.

[26] Lu J, Chu R, Yin Y, Yu H, Xu Q, Yang B (2022). Glycosylphosphatidylinositol-anchored micronemal antigen (GAMA) interacts with the band 3 receptor to promote erythrocyte invasion by malaria parasites. J Biol Chem.

[27] Shakya B, Penn WD, Nakayasu ES, LaCount DJ (2017). The Plasmodium falciparum exported protein PF3D7_0402000 binds to erythrocyte ankyrin and band 4.1. Mol Biochem Parasitol.

[28] Castro-Salguedo C, Mendez-Cuadro D, Moneriz C (2021). Erythrocyte membrane proteins involved in the immune response to *Plasmodium falciparum* and *Plasmodium vivax* infection. Parasitol Res.

[29] Wahlgren M, Goel S, Akhouri RR (2017). Variant surface antigens of *Plasmodium falciparum* and their roles in severe malaria. Nat Rev Microbiol.

[30] Tuikue Ndam N, Moussiliou A, Lavstsen T, Kamaliddin C, Jensen ATR, Mama A (2017). Parasites causing cerebral falciparum malaria bind multiple endothelial receptors and express EPCR and ICAM-1-binding PfEMP1. J Infect Dis.

[31] Matthews KM, Pitman EL, de Koning-Ward TF (2019). Illuminating how malaria parasites export proteins into host erythrocytes. Cell Microbiol.

[32] Wang B, Lu F, Cheng Y, Chen JH, Jeon HY, Ha KS (2015). Immunoprofiling of the tryptophan-rich antigen family in *Plasmodium vivax*. Infect Immun.

[33] Zeeshan M, Tyagi RK, Tyagi K, Alam MS, Sharma YD (2015). Host-parasite interaction: selective Pv-fam-a family proteins of *Plasmodium vivax* bind to a restricted number of human erythrocyte receptors. J Infect Dis.

[34] Rathore S, Dass S, Kandari D, Kaur I, Gupta M, Sharma YD (2017). Basigin interacts with *Plasmodium vivax* tryptophan-rich antigen PvTRAg38 as a second erythrocyte receptor to promote parasite growth. J Biol Chem.

[35] Zhang H, Shen F, Yu J, Ge J, Sun Y, Fu H (2022). *Plasmodium vivax* protein PvTRAg23 triggers spleen fibroblasts for inflammatory profile and reduces type I collagen secretion via NF-kappaBp65 pathway. Front Immunol.

[36] Bozdech Z, Mok S, Hu G, Imwong M, Jaidee A, Russell B (2008). The transcriptome of *Plasmodium vivax* reveals divergence and diversity of transcriptional regulation in malaria parasites. Proc Natl Acad Sci U S A.

[37] Zhang C, Gu Y, Tang J, Lu F, Cao Y, Zhou H (2016). Production of *Plasmodium vivax* enolase in *Escherichia coli* and its protective properties. Hum Vaccin Immunother.

[38] Briard D, Brouty-Boye D, Azzarone B, Jasmin C (2002). Fibroblasts from human spleen regulate NK cell differentiation from blood CD34(+) progenitors via cell surface IL-15. J Immunol.

[39] Kundu P, Naskar D, McKie SJ, Dass S, Kanjee U, Introini V (2023). The structure of a *Plasmodium vivax* tryptophan rich antigen domain suggests a lipid binding function for a pan-*Plasmodium* multi-gene family. Nat Commun.

[40] Innan H, Kondrashov F (2010). The evolution of gene duplications: classifying and distinguishing between models. Nat Rev Genet.

[41] Kar S, Sinha A (2022). *Plasmodium vivax* duffy binding protein-based vaccine: a distant dream. Front Cell Infect Microbiol.

[42] Lee WC, Russell B, Renia L (2019). Sticking for a cause: the falciparum malaria parasites cytoadherence paradigm. Front Immunol.

[43] Adams Y, Olsen RW, Bengtsson A, Dalgaard N, Zdioruk M, Satpathi S (2021). *Plasmodium falciparum* erythrocyte membrane protein 1 variants induce cell swelling and disrupt the blood-brain barrier in cerebral malaria. J Exp Med.

[44] Gowda DC, Wu X (2018). Parasite recognition and signaling mechanisms in innate immune responses to malaria. Front Immunol.

[45] Toda H, Diaz-Varela M, Segui-Barber J, Roobsoong W, Baro B, Garcia-Silva S (2020). Plasma-derived extracellular vesicles from *Plasmodium vivax* patients signal spleen fibroblasts via NF-kB facilitating parasite cytoadherence. Nat Commun.

[46] Kepple D, Ford CT, Williams J, Abagero B, Li S, Popovici J (2024). Comparative transcriptomics reveal differential gene expression among *Plasmodium vivax* geographical isolates and implications on erythrocyte invasion mechanisms. PLoS Negl Trop Dis.

[47] Neafsey DE, Galinsky K, Jiang RH, Young L, Sykes SM, Saif S (2012). The malaria parasite *Plasmodium vivax* exhibits greater genetic diversity than *Plasmodium falciparum*. Nat Genet.

[48] Gupta P, Das A, Singh OP, Ghosh SK, Singh V (2012). Assessing the genetic diversity of the vir genes in Indian *Plasmodium vivax* population. Acta Trop.

[49] Shen HM, Chen SB, Cui YB, Xu B, Kassegne K, Abe EM (2018). Whole-genome sequencing and analysis of *Plasmodium falciparum* isolates from China-Myanmar border area. Infect Dis Poverty.

[50] Tummino PJ, Copeland RA (2008). Residence time of receptor-ligand complexes and its effect on biological function. Biochemistry.

[51] Du R, Li L, Ji J, Fan Y (2023). Receptor-ligand binding: effect of mechanical factors. Int J Mol Sci.

[52] Zou W, Dong X, Broederdorf TR, Shen A, Kramer DA, Shi R (2018). A dendritic guidance receptor complex brings together distinct actin regulators to drive efficient F-actin assembly and branching. Dev Cell.

[53] Hong J, Zhang G, Dong F, Rechler MM (2002). Insulin-like growth factor (IGF)-binding protein-3 mutants that do not bind IGF-I or IGF-II stimulate apoptosis in human prostate cancer cells. J Biol Chem.

[54] Ollewagen T, Myburgh KH, van de Vyver M, Smith C (2021). Rheumatoid cachexia: the underappreciated role of myoblast, macrophage and fibroblast interplay in the skeletal muscle niche. J Biomed Sci.

[55] Tomlin H, Piccinini AM (2018). A complex interplay between the extracellular matrix and the innate immune response to microbial pathogens. Immunology.

[56] Hallmann R, Zhang X, Di Russo J, Li L, Song J, Hannocks MJ (2015). The regulation of immune cell trafficking by the extracellular matrix. Curr Opin Cell Biol.

[57] Kho S, Qotrunnada L, Leonardo L, Andries B, Wardani PAI, Fricot A (2021). Hidden biomass of intact malaria parasites in the human spleen. N Engl J Med.

[58] Kho S, Qotrunnada L, Leonardo L, Andries B, Wardani PAI, Fricot A (2021). Evaluation of splenic accumulation and colocalization of immature reticulocytes and *Plasmodium vivax* in asymptomatic malaria: a prospective human splenectomy study. PLoS Med.

[59] Fan J, Duan L, Wu N, Xu X, Xin J, Jiang S (2020). Baicalin ameliorates pancreatic fibrosis by inhibiting the activation of pancreatic stellate cells in mice with chronic pancreatitis. Front Pharmacol.

[60] Park MS, Kim YH, Lee JW (2010). FAK mediates signal crosstalk between type II collagen and TGF-beta 1 cascades in chondrocytic cells. Matrix Biol.

[61] Viale-Bouroncle S, Gosau M, Morsczeck C (2014). Collagen I induces the expression of alkaline phosphatase and osteopontin via independent activations of FAK and ERK signalling pathways. Arch Oral Biol.

[62] Kang P, Wang J, Fang D, Fang T, Yu Y, Zhang W (2020). Activation of ALDH2 attenuates high glucose induced rat cardiomyocyte fibrosis and necroptosis. Free Radic Biol Med.

