# Supplementary material for: Plasmodium vivax tryptophan-rich antigen reduces type I collagen secretion via the NF-κBp65 pathway in splenic fibroblasts
Source: Parasit Vectors. 2024 May 27;17:239. doi: 10.1186/s13071-024-06264-y (PMC11131192; doi:10.1186/s13071-024-06264-y)
Supplement: Supplementary file 1 — Additional file 1: Table S1. Primers used for the amplification of pvtrag genes. [file 13071_2024_6264_MOESM1_ESM.docx]

| **Table S1** Primers used for the amplification of *pvtrag* genes. | | | | |  |
| --- | --- | --- | --- | --- | --- |
| Name | PvP01 Gene ID | PvSal I Gene ID | Forward primer (5' → 3')*^a^* | Reverse primer (5' → 4')*^a^* |  |
| TRAg_1 | PVP01_0201100 | PVX_096950 | gctgatatcggatccAGCGCCAGAGACCAAT | gtggtggtgctcgagTAAGCACTGTTGGAGGAAGAAG |  |
| TRAg_2 | PVP01_0202200 | PVX_096995 | gctgatatcggatccAAAACCGAAACGGTG | gtggtggtgctcgagTTCCTCTTCTTTCTTTTCGGG |  |
| TRAg_3 | PVP01_0404200 | PVX_002500 | gctgatatcggatccTCTCTTCAATATGAACAAATTGAACCC | gtggtggtgctcgagGTATTTATTATTTTTCCATTCTGACCAAT |  |
| TRAg_4 | PVP01_0503400 | PVX_088810 | gctgatatcggatccAACCCCTTGAGTGGCG | gtggtggtgctcgagTTCTGCATTCAGTGGGGC |  |
| TRAg_5 | PVP01_0503600 | PVX_088820 | gctgatatcggatccGCGGCAGCTAATAGACCAA | gtggtggtgctcgagAGTTGACCATTTGTTAAATAGAATTTTT |  |
| TRAg_6 | PVP01_0503700 | PVX_088825 | gctgatatcggatccTTTCTCCCCACACTTGGA | gtggtggtgctcgagAGTAAACATTTCCACCCATTTAC |  |
| TRAg_7 | PVP01_0504200 | PVX_088850 | gctgatatcggatccTTCTTCAGTAAAAAGTCGAACAGA | gtggtggtgctcgagTTCAAAGAATTCAAATGCGAAAGC |  |
| TRAg_8 | PVP01_0532600 | PVX_090250 | gctgatatcggatccATGATGGCCACCCAGATATAC | gtggtggtgctcgagCTTGACCCAGACGAACCA |  |
| TRAg_9 | PVP01_0532700 | PVX_090255 | gctgatatcggatccTCCGCCAAAAAAAAAGCCAAA | gtggtggtgctcgagTTGGTTGGAGTAATGCTTCCAC |  |
| TRAg_10 | PVP01_0532800 | PVX_090260 | gctgatatcggatccTCCGTTAATAAGAAAAAGAAGGACAT | gtggtggtgctcgagCATCTGCGGGTCTCCC |  |
| TRAg_11 | PVP01_0532900 | PVX_090265 | gctgatatcggatccCATGGAGATATCAATGACTTCCTAGGA | gtggtggtgctcgagTTTTTTTTTATTTAATGCAGCTTTCGCC |  |
| TRAg_12 | PVP01_0533000 | PVX_090270 | gctgatatcggatccTCCATACAAGTGAAAACGAACG | gtggtggtgctcgagCTCCTTAAGCCACACTTGC |  |
| TRAg_13 | PVP01_0533100 | PVX_090275 | gctgatatcggatccGCTGCCCAAAAGAAGGC | gtggtggtgctcgagTTTATCATTTTTCCATTTGGTCCATG |  |
| TRAg_14 | PVP01_0801800 | PVX_094305 | gctgatatcggatccAACTCCTTCAGAAAAAGGGGACA | gtggtggtgctcgagTTTTTTCCCCCCTGGCG |  |
| TRAg_15 | PVP01_0948700 | PVX_092990 | gctgatatcggatccAACGATTTAGAATTGGAAAATGCT | gtggtggtgctcgagAAATGTTGTACACATTGAATTGAACACA |  |
| TRAg_16 | PVP01_0948800 | PVX_092995 | gctgatatcggatccAGATGGTTTACCTTCACTTCTC | gtggtggtgctcgagCGTATTCCATTGTTTGTCTTGT |  |
| TRAg_17 | PVP01_1033800 | PVX_097577 | gctgatatcggatccGCAAGCACCGAAAGGG | gtggtggtgctcgagTGAGTCATTATCTGTGCTCACC |  |
| TRAg_18 | PVP01_1033900 | PVX_097575 | gctgatatcggatccTTTCCTCGTGATCATAGAGCG | gtggtggtgctcgagAAAGTGGTGGAGCATAGAGA |  |
| TRAg_19 | PVP01_1101400 | PVX_115465 | gctgatatcggatccTCGATAGAGAGGCAGCAAGA | gtggtggtgctcgagTATCCATTTTTCAATAAACGGCTTCA |  |
| TRAg_20 | PVP01_1201800 | PVX_083550 | gctgatatcggatccATCCTGCAGCCGCG | gtggtggtgctcgagTTCCAGTTTTACGTTTGATTCGTAGA |  |
| TRAg_21 | PVP01_1401800 | PVX_121897 | gctgatatcggatccTCCTCTCAAAGCGCAGTAGA | gtggtggtgctcgagTCTCCAGGTGCTGTATAAGG |  |
| TRAg_22 | PVP01_1469800 | PVX_101510 | gctgatatcggatccCCAATGCTTCAACATGCAAAAG | gtggtggtgctcgagCTGCTTCCTGCTGATCCAT |  |
| TRAg_23 | PVP01_1469900 | PVX_101515 | gctgatatcggatccAAAAAAAATGATAATATACCACAGCTCC | gtggtggtgctcgagTACCCACGTGTTGATAAATGATTCT |  |
| TRAg_24 | PVP01_1470100 | PVX_101525 | gctgatatcggatccTCATCTCACCATGGAAGGTG | gtggtggtgctcgagAAAGTCGTTTCTCCAATTATTAAATAAAAAGT |  |
| TRAg_25 | PVP01_0000100 | PVX_112655 | gctgatatcggatccTCTGTATTCAAAAAATTAAATAACACATTA | gtggtggtgctcgagTATCCATTTGTTAATGAAGGTCAAAAACT |  |
| TRAg_26 | PVP01_0000110 | PVX_112660 | gctgatatcggatccCCTTCACTTATAGATAAGTACGATGCT | gtggtggtgctcgagCTTCCACTGAATCCATTTTTTCAAA |  |
| TRAg_27 | PVP01_0000120 | PVX_112665 | gctgatatcggatccGATGCTCAGGGTGAGAACG | gtggtggtgctcgagTGTCCATTTTTCAACAAAATATTCCA |  |
| TRAg_28 | PVP01_0000130 | PVX_112670 | gctgatatcggatccGAAGCTGAAGTAACCGAAGGT | gtggtggtgctcgagTGCTTTCTTTTCCCAGTTTTTG |  |
| TRAg_29 | PVP01_0000140 | PVX_112675 | gctgatatcggatccCAAGCTTTGCCAAAACCTG | gtggtggtgctcgagATAATTACTCCACATATTATCCAAATTATTCG |  |
| TRAg_30 | PVP01_0000150 | PVX_112680 | gctgatatcggatccCAAGCTGTACCCAAACCTGA | gtggtggtgctcgagTTCTTCTATCCATTCTTTAAATCGAAAGTA |  |
| TRAg_31 | PVP01_0000160 | PVX_112685 | gctgatatcggatccGAAGCTGGCAAGAAACCTG | gtggtggtgctcgagAAATAATGTATCTTTTTGATAAGCCTTCC | |
| TRAg_32 | PVP01_0000170 | PVX_112690 | gctgatatcggatccGAAGCTATGCCCAAATTTCCTCAG | gtggtggtgctcgagTTGTGGAGCCCATTCATCAAAG | |
| TRAg_33 | PVP01_0000200 | PVX_112705 | gctgatatcggatccATGAAAACGAGGGGCTATCAA | gtggtggtgctcgagTGTGTTTTCCTTCCAATTTTCAAACA | |
| TRAg_34 | PVP01_0700700 | PVX_125728 | gctgatatcggatccAAATCAAGCAACGAAATTGAAAGAC | gtggtggtgctcgagGGAGTCCATCCATGTTTGAAAG | |
| TRAg_35 | PVP01_0700800 | PVX_125730 | gctgatatcggatccACCGTTTTAGAACCAGTGGAAG | gtggtggtgctcgagAACGAACATATTCCATTTTTTTTCG | |
| TRAg_36 | PVP01_0949200 | PVX_109280 | gctgatatcggatccTCCCTTCAAAATGAATGCAACA | gtggtggtgctcgagCCATTTTTCCTCACGTATCCAATT | |

^a^ Lowercase letter of primer sequence indicates it is homologous to the vector sequence.
