# Supplementary material for: Plasmodium vivax tryptophan-rich antigen reduces type I collagen secretion via the NF-κBp65 pathway in splenic fibroblasts
Source: Parasit Vectors. 2024 May 27;17:239. doi: 10.1186/s13071-024-06264-y (PMC11131192; doi:10.1186/s13071-024-06264-y)
Supplement: Supplementary file 2 — Additional file 2: Table S2. Sequences of the primers used for inflammatory factors. [file 13071_2024_6264_MOESM2_ESM.docx]

**Table S2**  Sequences of the primers used for .inflammatory factors.

| Gene name | Primer forward (5’−3’) | Primer Reverse (3’−5’) |
| --- | --- | --- |
| IL-1β | ATGATGGCTTATTACAGTGGCAA | GTCGGAGATTCGTAGCTGGA |
| IL-6 | CCTGAGAAAGGAGACATGTAACAA | GGCAAGTCTCCTCATTGAATCC |
| TNF-α | CCCAGGCAGTCAGATCATCTTCT | ATGAGGTACAGGCCCTCTGAT |
| GAPDH | CCTGCCTCTACTGGCGCTGC | GCAGTGGGGACACGGAAGGC |
