# Supplementary material for: Plasmodium vivax tryptophan-rich antigen reduces type I collagen secretion via the NF-κBp65 pathway in splenic fibroblasts
Source: Parasit Vectors. 2024 May 27;17:239. doi: 10.1186/s13071-024-06264-y (PMC11131192; doi:10.1186/s13071-024-06264-y)
Supplement: Supplementary file 3 — Additional file 3: Table S3. Characterization information for all members of the PvTRAg family. [file 13071_2024_6264_MOESM3_ESM.docx]

| **Table S3** Characterization information for all members of the PvTRAg family. | | | | | | | | | | |
| --- | --- | --- | --- | --- | --- | --- | --- | --- | --- | --- |
| Name | PvP01 Gene ID | PvSalI Gene  ID | Protein  Length | Isoelectric  Point | Molecular  Weight | Signal  Peptide | Transmembrane  Domains | Tryptophan-rich  Domain (aa) | Glycophosphatidylinositol  (GPI)-anchored |  |
| TRAg_1 | PVP01_0201100 | PVX_096950 | 327 | 9.92 | 39897 | 0 - 28 | no | 99 - 316 | 311 - 327 |  |
| TRAg_2 | PVP01_0202200 | PVX_096995 | 480 | 4.52 | 55024 | no | 32 - 55 | 93 - 308 | 454 - 480 |  |
| TRAg_3 | PVP01_0404200 | PVX_002500 | 276 | 4.41 | 33694 | 0 - 26 | no | 58 - 269 | 248 - 276 |  |
| TRAg_4 | PVP01_0503400 | PVX_088810 | 358 | 4.79 | 42933 | no | no | 91 - 307 | 327 - 358 |  |
| TRAg_5 | PVP01_0503600 | PVX_088820 | 316 | 10.29 | 38021 | no | 34 - 57 | 90 - 306 | 292 - 316 |  |
| TRAg_6 | PVP01_0503700 | PVX_088825 | 444 | 10.05 | 53743 | no | 36 - 62 | 225-441 | 430 - 444 |  |
| TRAg_7 | PVP01_0504200 | PVX_088850 | 457 | 4.8 | 56270 | no | 39 - 62 | 177 - 393 | 439 - 457 |  |
| TRAg_8 | PVP01_0532600 | PVX_090250 | 322 | 8.06 | 39810 | no | no | 90 - 305 | 308 - 322 |  |
| TRAg_9 | PVP01_0532700 | PVX_090255 | 326 | 5 | 39916 | no | 35 - 58 | 93 - 308 | 313 - 326 |  |
| TRAg_10 | PVP01_0532800 | PVX_090260 | 346 | 9.49 | 42925 | no | 37 - 60 | 92 - 309 | 333 - 346 |  |
| TRAg_11 | PVP01_0532900 | PVX_090265 | 326 | 6.54 | 40143 | no | 35 - 58 | 90 - 307 | 314 - 326 |  |
| TRAg_12 | PVP01_0533000 | PVX_090270 | 422 | 10.28 | 50253 | 0 - 23 | no | 178 - 396 | 400 - 422 |  |
| TRAg_13 | PVP01_0533100 | PVX_090275 | 339 | 10.2 | 38812 | 0 - 24 | no | 119 - 338 | 315 - 339 |  |
| TRAg_14 | PVP01_0801800 | PVX_094305 | 869 | 10.02 | 99688 | no | no | 612 - 825 | 847 -869 |  |
| TRAg_15 | PVP01_0948700 | PVX_092990 | 1414 | 3.36 | 157511 | 0 - 21 | 1 - 21 | 1192 - 1408 | 1396 - 1414 |  |
| TRAg_16 | PVP01_0948800 | PVX_092995 | 358 | 10.1 | 42999 | 0 - 23 | no | 75 - 289 | 337 - 358 |  |
| TRAg_17 | PVP01_1033800 | PVX_097577 | 315 | 5.81 | 38760 | no | 31 - 54 | 92 - 307 | 296 - 315 |  |
| TRAg_18 | PVP01_1033900 | PVX_097575 | 2662 | 5.6 | 309186 | no | 31 - 51 | 2436 - 2651 | 2639 - 2662 |  |
| TRAg_19 | PVP01_1101400 | PVX_115465 | 598 | 3.99 | 70546 | no | 303 - 326 | 369 - 585 | 578 - 598 |  |
| TRAg_20 | PVP01_1201800 | PVX_083550 | 612 | 4.8 | 73453 | no | no | 372 - 583 | 599 - 612 |  |
| TRAg_21 | PVP01_1401800 | PVX_121897 | 275 | 4.7 | 33504 | 0 - 15 | no | 56 - 272 | 258 - 275 |  |
| TRAg_22 | PVP01_1469800 | PVX_101510 | 715 | 5.1 | 82287 | no | 29 - 52 | 483 - 699 | 696 - 715 |  |
| TRAg_23 | PVP01_1469900 | PVX_101515 | 321 | 9.51 | 39951 | no | 35 - 55 | 95 - 311 | 304 - 321 |  |
| TRAg_24 | PVP01_1470100 | PVX_101525 | 287 | 4.59 | 35729 | no | no | 67 - 281 | 269 - 287 |  |
| TRAg_25 | PVP01_0000100 | PVX_112655 | 692 | 6.95 | 80685 | no | no | 467 - 683 | 676 - 692 |  |
| TRAg_26 | PVP01_0000110 | PVX_112660 | 223 | 4.31 | 26328 | no | no | 93 - 211 | 206 - 223 |  |
| TRAg_27 | PVP01_0000120 | PVX_112665 | 288 | 6.37 | 35254 | 0 - 28 | no | 61 - 278 | 271 - 288 |  |
| TRAg_28 | PVP01_0000130 | PVX_112670 | 335 | 6.1 | 38528 | no | no | 143 - 272 | 306 - 335 |  |
| TRAg_29 | PVP01_0000140 | PVX_112675 | 312 | 9.09 | 36026 | 0 - 31 | 6 - 26 | 121 - 247 | 279 - 312 |  |
| TRAg_30 | PVP01_0000150 | PVX_112680 | 313 | 10.13 | 36755 | 0 - 28 | no | 123 - 245 | 294 - 313 |  |
| TRAg_31 | PVP01_0000160 | PVX_112685 | 302 | 9.51 | 34088 | 0 - 28 | no | 119 - 230 | 282 - 302 |  |
| TRAg_32 | PVP01_0000170 | PVX_112690 | 313 | 9.1 | 36606 | no | 6 - 29 | 122 - 248 | 294 - 313 |  |
| TRAg_33 | PVP01_0000200 | PVX_112705 | 294 | 8.96 | 37499 | no | no | 97 - 281 | 269 - 294 |  |
| TRAg_34 | PVP01_0700700 | PVX_125728 | 279 | 6.19 | 34921 | 0 - 20 | no | 59 - 274 | 262 - 279 |  |
| TRAg_35 | PVP01_0700800 | PVX_125730 | 330 | 6.13 | 40826 | no | no | 92 - 307 | 309 - 330 |  |
| TRAg_36 | PVP01_0949200 | PVX_109280 | 283 | 7.03 | 35266 | 0 - 19 | no | 56 - 271 | 270 - 283 |  |
|  | PVP01_0700900 | no | 298 | 8.17 | 36996 | 0 - 32 | no | 65-285 | no |  |
|  | PVP01_0949000 | no | 334 | 10.02 | 40433 | 0 - 20 | no | 77 - 287 | no |  |
|  | PVP01_0948900 | - | 333 | 10.15 | 39811 | 0 - 20 | no | 85 - 281 | no |  |
|  | PVP01_0000210 | - | 256 | 7.36 | 33291 | no | no | 43 - 251 | no |  |

aa：Amino acids.
